# Neighborhood Danger, Parental Monitoring, Harsh Parenting, and Child Aggression in Nine Countries

**DOI:** 10.3390/soc4010045

**Published:** 2014-01-20

**Authors:** Ann T. Skinner, Dario Bacchini, Jennifer E. Lansford, Jennifer Godwin, Emma Sorbring, Sombat Tapanya, Liliana Maria Uribe Tirado, Arnaldo Zelli, Liane Peña Alampay, Suha M. Al-Hassan, Anna Silvia Bombi, Marc H. Bornstein, Lei Chang, Kirby Deater-Deckard, Laura Di Giunta, Kenneth A. Dodge, Patrick S. Malone, Maria Concetta Miranda, Paul Oburu, Concetta Pastorelli

**Affiliations:** 1 Center for Child and Family Policy, Duke University, Durham, NC 27708 USA; 2 Department of Psychology, Second University of Naples, 81100 Caserta, Italy; 3 Department of Psychology, University West, 46186 Trollhättan, Sweden/emma.sorbring@hv.se; 4 Department of Psychiatry, Chiang Mai University, Chiang Mai 50200, Thailand/ sombat.tapanya@gmail.com; 5 Consultorio Psicológico Popular, Universidad San Buenaventura, Medellín, Colombia/lilianauribe74@gmail.com; 6 Department of Education Sciences, “Foro Italico”, University of Rome, 00135 Rome, Italy/arnaldo.zelli@uniroma4.it; 7 Department of Psychology, Ateneo de Manila University, Quezon City 1108, Philippines/lpalampay@ateneo.edu; 8 Queen Rania Faculty for Childhood, The Hashemite University, Zarqa 13115, Jordan/suha_al@yahoo.com; 9 Faculty of Psychology, Università di Roma “La Sapienza”, 00185 Rome, Italy/annasilvia.bombi@uniroma1.it; 10 Child and Family Research Program in Developmental Neuroscience, Eunice Kennedy Shriver National Institute of Child Health and Human Development, Bethesda, MD 20892, USA/Marc_H_Bornstein@nih.gov; 11 Department of Educational Psychology, The Chinese University of Hong Kong, Shatin, Hong Kong/leichang@cuhk.edu.hk; 12 Department of Psychology, Virginia Polytechnic Institute and State University, Blacksburg, VA 24061, USA/kirbydd@vt.edu; 13 Department of Psychology, University of South Carolina, Colombia, SC 29208 USA/malone.ps@gmail.com

**Keywords:** child aggression, community violence, harsh parenting, parental monitoring, neighborhood danger

## Abstract

Exposure to neighborhood danger during childhood has negative effects that permeate multiple dimensions of childhood. The current study examined whether mothers’, fathers’, and children's perceptions of neighborhood danger are related to child aggression, whether parental monitoring moderates this relation, and whether harsh parenting mediates this relation. Interviews were conducted with a sample of 1,293 children (age *M* = 10.68, *SD* = .66; 51% girls) and their mothers (*n* = 1,282) and fathers (*n* = 1,075) in nine countries (China, Colombia, Italy, Jordan, Kenya, the Philippines, Sweden, Thailand, and the United States). Perceptions of greater neighborhood danger were associated with more child aggression in all nine countries according to mothers’ and fathers’ reports and in five of the nine countries according to children's reports. Parental monitoring did not moderate the relation between perception of neighborhood danger and child aggression. The mediating role of harsh parenting was inconsistent across countries and reporters. Implications for further research are discussed, and include examination of more specific aspects of parental monitoring as well as more objective measures of neighborhood danger.

## 1. Introduction

Childhood exposure to community violence has a negative impact on child adjustment. Samples drawn primarily from urban areas in the United States link exposure to community violence with children's internalizing symptoms [1], academic difficulties [2] and aggressive behavior [3-5]. For example, in a study with 97 6- to 10-year-old American at-risk youth in an urban setting, child-reported exposure to community violence predicted changes in parent-reported antisocial behavior [6]. Similar links between community violence exposure and child behavior problems have been found in other countries as well [7, 8].

Early research on children's exposure to community violence focused on identifying prevalence rates and documenting direct effects. Recent research has begun to examine mediators and moderators of links between exposure to community violence and child adjustment, particularly aggression [9]. The current study helps to fill a gap in the literature in two ways. First, the link between community violence, as measured by perception of neighborhood danger, and child aggression is examined in nine countries (China, Colombia, Italy, Jordan, Kenya, the Philippines, Sweden, Thailand, and the United States). Second, in these same countries, parental monitoring and harsh parenting are examined as moderating and mediating variables, respectively.

### 1.1 Neighborhood Danger and Child Aggression

The relation between exposure to neighborhood danger and child aggression is well-established in the literature. In communities where neighborhood violence is a chronic problem, exposure to such violence has been linked to numerous externalizing behavior outcomes for children. In a sample of 11-12 year old American youth from high-poverty communities, higher rates of witnessing violence were related to increased intention to use violence [10]. Other research shows a relation between exposure to community violence and aggressive behavior, even when controlling for previous levels of aggression [3]. Furthermore, the effects appear to linger beyond childhood; in a sample of young adults who were asked to report about their lifetime exposure to violence, those in the high-exposure group reported greater levels of aggression [11]. While much of extant literature documenting the relation between child aggression and neighborhood danger includes samples from the United States, and with immigrant samples within the United States [7], little research is available documenting the effects in other countries. One example, with a sample from Italy, documented increases in antisocial behavior (including theft, use of drugs, fighting and stealing) with exposure to community violence [12]. Further research in other countries is needed to see if the strong link between neighborhood danger and child aggression found in samples from the United States holds in other countries.

### 1.2 Parental Monitoring

Although previous research has demonstrated that exposure to neighborhood danger is related to children's aggression, this link may not hold in all circumstances or for all children. For example, parents may behave in ways that attenuate the risk of exposure to neighborhood danger. In particular, parents in dangerous neighborhoods may make themselves aware of their children's activities, whereabouts, and friends in ways that serve a protective function [13, 14]. Thus, parental monitoring may moderate the association between exposure to neighborhood danger and children's aggression if this link holds (or holds more strongly) for children whose parents provide low levels of monitoring compared to children whose parents provide high levels of monitoring.

Previous research has been inconclusive regarding whether parental monitoring moderates the relation between exposure to community violence and child aggression. In a confirmation of previous work [3], Bacchini, Miranda, and Affuso [12]found that in a sample of 489 adolescents in Naples, Italy, high levels of parental monitoring moderated the relation between exposure to low levels of community violence and antisocial behavior. That is, one negative effect of exposure to community violence (i.e., antisocial behavior) was not as strong when high levels of parental monitoring were present under conditions of low levels of community violence. Similarly, in a study that examined the relation between witnessing violence and subsequent substance use, parental monitoring played a moderating role, but only at low levels of witnessing violence[14]. Witnessing violence predicted future initiation of substance use, but high levels of parental monitoring were related to a lower likelihood of initiation of use of cigarettes and liquor. Other research [15]found that high levels of residential mobility were more strongly related to externalizing behavior at age 11 when parents monitored less.

Although these studies are mixed with regard to the moderating role of parental monitoring in the relation between exposure to neighborhood danger and youth aggression, taken together, they suggest two points. First, because previous studies did not consistently take into account the role of the parents’ and child's neighborhood, an ecological approach [16] is warranted to examine the effects of neighborhood danger on child aggression in the context of family processes. Second, because parents may underestimate a child's negative experience with neighborhood danger, both child and parent perceptions of neighborhood danger are important. Both points are addressed by this study.

### 1.3 Harsh parenting

Conceptually, as parental monitoring is a leading contender as a potential moderator of the link between exposure to neighborhood danger and children's aggression, harsh parenting is a leading contender as a potential mediator of this association. That is, a primary mechanism through which neighborhood danger affects children's aggression may be through harsh parenting. Harsh parenting can encompass both verbal and physical dimensions and has been operationalized most frequently in terms of corporal punishment. Corporal punishment is consistently related to more child aggression both in the United States [17]and in a number of other countries [18, 19]. A study of 100 Kenyan children, for example, revealed that childrearing violence in one year predicted child externalizing behavior one year later [20]. Similarly, in a study with 9,705 Finnish and Danish children, more severe harsh parenting, characterized by behaviors such as hitting, hitting with an object, kicking, and using or threatening to use a knife or a gun, was related to both internalizing and externalizing symptoms in youth [21].

When mothers are exposed to high levels of community violence, they are more likely to engage in physically aggressive parenting compared to mothers with no such exposure to community violence [22]. For example, in the United States, child maltreatment is higher in disadvantaged neighborhoods, and those likely to be perceived as more dangerous [23]. This relation has been found in several other countries as well. Using data from 186 cultural groups, Lansford and Dodge [24] found that parents who were living among other forms of violence were also more likely to use corporal punishment on their children. It follows, then, that harsh parenting may also play a role in the relation between exposure to neighborhood danger and child aggression.

### 1.4 Measuring neighborhood danger

Definitions and methodology are important considerations in the study of neighborhood danger and child adjustment. There are both objective (e.g., measuring crime rates) and subjective (e.g., asking respondents for their perceptions about violence in their community) ways to measure neighborhood danger. Although many studies have relied on single-reporter data to measure neighborhood violence, this proves to be inadequate. For example, gathering information only from parents can lead to underestimating the danger to which children are exposed in the neighborhood, and thus also underestimating the effects on children [25]. Neither is it wise to eliminate parents’ perceptions, as their perceptions drive the very behavior we aim to study [26], specifically parental monitoring and harsh parenting. In one sense, perceptions are even more important than actual rates of violence, because reality may be irrelevant in the face of what parents perceive as threats to themselves and their children. Other researchers have found that children's perceptions of living in a dangerous neighborhood – rather than actual rates of violent incidents children were exposed to – more accurately predicted the relation between exposure to neighborhood violence and child adjustment [27]. For these reasons, our study includes perceptions of neighborhood danger as the variable measuring community violence.

### 1.5 The Present Study

In an analysis of the sample characteristics in the most influential journals in six sub-disciplines of psychology from 2003-2007, 96% of research participants were from Western industrialized countries, and 68% were from the United States alone [28]. This finding means that 96% of research participants in these psychological studies were from countries with only 12% of the world's population [29]. Findings from these limited samples may not generalize to more diverse populations [29]. To advance understanding of community violence in diverse countries around the world, we developed the Parenting Across Cultures project as an international collaboration among nine countries: China, Colombia, Italy, Jordan, Kenya, the Philippines, Sweden, Thailand, and the United States. This sample of countries was diverse on several socio-demographic dimensions, including predominant ethnicity, predominant religion, economic indicators, and indices of child well-being. For example, on the Human Development Index, a composite indicator of a country's status with respect to health, education, and income, participating countries ranged from a rank of 4 to 128 out of 169 countries with available data. To provide a sense of what this range entails, the infant mortality rate in Kenya, for example, is 40 times higher than the infant mortality rate in Sweden. In the Philippines, 23% of the population falls below the international poverty line of less than US$1.25 per day, whereas none of the population falls below this poverty line in Italy, Sweden, or the United States. The participating countries varied widely not only on socio-demographic indicators, but also on psychological constructs such as individualism versus collectivism. Using Hofstede's [30] rankings, the participating countries ranged from the United States, with the highest individualism score in the world to China, Colombia, and Thailand, countries that are among the least individualistic in the world. The purpose of recruiting families from these countries was to create an international sample that would be diverse with respect to a number of socio-demographic and psychological characteristics. Ultimately, this diversity provided us with an opportunity to examine our research questions in a sample that is more generalizable to a wider range of the world's populations than is typical in most research to date.

In the present study, we examined three research questions. First, is the perception of living in a dangerous neighborhood associated with more child aggression in a variety of countries? We hypothesized that, similar to studies documenting the effect of community violence in the United States, we would find evidence that living in a neighborhood with more perceived danger predicts child aggression in other countries, as well. Although community violence and perceptions of neighborhood danger could be quite different conceptually, we wanted to strengthen our study by including both parent and child perceptions of neighborhood danger. Second, is the link between perceived neighborhood danger and child aggression moderated by parental monitoring, and if so, is that moderating role consistent across countries? We hypothesized that parental monitoring would moderate the relation between perceptions of neighborhood danger and child aggression across countries. Third, is the link between neighborhood danger and child aggression mediated by harsh parenting, and if so, is that link consistent across countries? Again, we hypothesized that in all countries, harsh parenting would mediate the link between perceptions of neighborhood danger and child aggression.

## 2. Method

### 2.1 Participants

Participants included 1,293 children (age *M* = 10.68, *SD* = .66; 51% girls) and their mothers (*n* = 1,282) and fathers (*n* = 1,075). Families were drawn from Jinan and Shanghai, China (*n* = 218), Medellín, Colombia (*n* = 100), Naples and Rome, Italy (*n* = 194), Zarqa, Jordan (*n* = 112), Kisumu, Kenya (*n* = 95), Manila, Philippines (*n* = 103), Trollhättan/Vänersborg, Sweden (*n*=98), Chiang Mai, Thailand (*n* = 101), and Durham, North Carolina, United States (*n* = 272). In Sweden, the survey instrument for children did not include the neighborhood danger items; therefore, Sweden was omitted from the analyses based on child-reported data.

Children were recruited through schools representing a diverse range of socioeconomic backgrounds in each country. Letters describing the study were sent home with children, and parents were asked to return a signed form if they were willing to be contacted about the study (in some countries) and contacted by phone to follow up on the letter (in other countries). Families were then enrolled in the study until the target sample size (*n* = 100) was reached in each country. To make each country's sample as representative as possible of the city from which it was drawn, families of students from private and public schools were recruited in the approximate proportion to which they were represented in the population of the city. Furthermore, children were sampled from schools serving high-, middle-, and low-income families in the approximate proportion to which these income groups were represented in the local population. These sampling procedures resulted in an economically diverse sample that ranged from low income to high income within each site. The measures for the present analyses were from the third annual wave of data collection after recruitment; during this wave of data collection, 90.3% of children, 89.5% of mothers, and 75.1% of fathers completed surveys.

A procedure of forward- and back-translation ensured the linguistic and conceptual equivalence of all measures[31-33]. Interviews were conducted in participants’ homes, schools, or at another location chosen by the parents and used oral and written methods as appropriate. Mothers, fathers, and children were interviewed separately so that they could not hear one another's responses. Children were given small gifts in appreciation of their participation, and parents were given modest financial compensation for their participation, families were entered into drawings for prizes, or modest financial contributions were made to children's schools.

### 2.2 Measures

#### 2.2.1 Child Aggression

Child aggression was measured using Achenbach's [34] Child Behavior Checklist (CBCL) completed by parents and the Youth Self Report (YSR) completed by the child participants. The Achenbach measures have been translated into at least 69 languages, and over 5,000 published studies have used this measure with at least 60 cultural groups [35]. Aside from the measures’ widespread use in different countries [36], several researchers have specifically demonstrated good psychometric properties and cross-cultural and cross-language equivalence of the measures across cultural groups [37]. Parent-reported aggression was measured by 20 items, and child reports included 19 items. These items captured behaviors such as argues a lot, screams a lot, is disobedient at home, and threatens people. Each item measured the frequency a child participated in a particular behavior: *never* (coded as 0), *sometimes* (coded as 1), or *often* (coded as 2). For each reporter, a child aggression scale was created by averaging across these items.

#### 2.2.2 Neighborhood Danger

Neighborhood danger was assessed by 4 items reported by mothers, fathers, and children. The four possible responses ranged from *never true* (coded as 0) to *always true* (coded as 3). The items captured whether the respondent feels scared in the neighborhood, believes that many neighborhood children get into trouble, believes there are lots of drugs and gangs in the neighborhood, and feels the neighborhood is a dangerous place to live. For each reporter, a four-item mean score was created to capture perceptions of neighborhood danger. Although this measure has not been used widely in diverse countries, examination of the alpha coefficients in this sample (Table 2) suggests acceptable reliability. Rather than include reports of victimization or witnessing of violence, this method for assessing perception of neighborhood danger based on levels of gang activity and level of general danger has been used in other studies that linked perceptions of neighborhood danger to poorer social skills of children [26] and child aggression [38].

#### 2.2.3 Parental Monitoring

Respondents reported on ten items derived from Conger, Ge, Elder, Lorenz, and Simons [39] and Steinberg, Dornbusch, and Brown [40] to assess parental monitoring. The first five items captured how much the parent tries to gain knowledge about different activities in which the child participates (i.e., with whom the child spends time, how the child spends his/her free time, how the child spends his/her money, where the child goes right after school, and the type of homework the child receives). The three possible responses included: I *do not try* (coded as 0), I *try a little* (coded as 1), and I *try a lot* (coded as 2). The last five items captured the frequency with which the parent imposes limits on the child's activities (i.e., with whom the child spends time, how the child spends his/her free time, how the child spends his/her money, where the child goes right after school, and homework). The four possible responses ranged from *never* (coded as 0) to *always* (coded as 3). For each reporter, these items were standardized, and a parental monitoring scale was created by averaging across the standardized items. Several studies have measured parental monitoring validly and reliably in this way, including measures of both awareness/supervision and attempts to limit child behavior [12, 41, 42].

#### 2.2.4 Harsh Parenting

Harsh parenting was assessed using parent reports on items developed by UNICEF [43] for their Multiple Indicator Cluster Survey. UNICEF selected the items by convening an international panel of 25 experts to identify candidate items from existing valid and reliable measures of caregiving; field testing candidate items via cognitive interviews and quantitative surveys in the Americas, South Asia, and Africa; and convening a second international panel of 27 experts to evaluate items’ performance within and across diverse cultures and settings [44]. The items that resulted from this process were adapted from the Parent-Child Conflict Tactics Scale [45] and the WorldSAFE survey questionnaire [46]. The measure included seven yes/no items capturing whether the parent engaged in each of the following behaviors: shaking, shouting, spanking with a bare hand, hitting with a belt, calling the child dumb or lazy, slapping on the face, and slapping on the hand. Thus, our harsh parenting construct included both corporal punishment and harsh verbal responses to the child. For each parent, a harsh parenting scale was created by averaging the 7 items. Because children did not complete this measure, the average of the father and mother scores was used as the measure of harsh parenting in the child models.

### 2.3 Analytic Approach

For each reporter (father, mother, and child), the relation between neighborhood danger and child aggression was assessed using a multiple group path analysis framework to account for differences in the nine countries. This initial model also included the main effects of the potential moderator (parental monitoring) and mediator (harsh parenting). These estimates are included in the figures but are not discussed in the text as these relations are not included in our hypotheses for this study. All scales were grand mean-centered prior to inclusion in the models. Full-information maximum likelihood estimation was used to account for missing data. Chi-square tests using Satorra-Bentler [47] scaled chi-square estimates were calculated to determine whether the model fit improved when each relation was allowed to vary by country. If fit did improve, pairwise tests comparing the differences in the relation between all countries were conducted using Holm's correction for multiple post-hoc comparisons [48].

We examined whether parental monitoring moderated the relation between neighborhood danger and child aggression by including the interaction between the parental monitoring and danger scales. Finally, to examine whether harsh parenting mediated the relation between neighborhood danger and child aggression, we allowed neighborhood danger to predict harsh parenting. The indirect effect of neighborhood danger on aggression through harsh parenting was then estimated as the product of the harsh parenting/neighborhood danger relation and the neighborhood danger and child aggression relation.

Models reported here do not include additional demographic control variables. However, all models were also estimated controlling for child's gender, child's age, years of formal education of the most educated parent, and family income. Table 1 provides descriptive statistics for these variables by country. Income was reported in each of the countries using a list of income levels representing 10 divisions of income in local currency with a value of “1” corresponding to the lowest level of income, and “10” corresponding to the highest, except in Jordan, where the income range was 1-5. The results did not change when these additional control variables were included.

## 3. Results

### 3.1 Descriptive Statistics

Table 2 describes the means and standard deviations of the scales within each country, as well as the Cronbach's alpha coefficients. For each scale, analysis of variance models revealed statistically significant differences in means across countries. Across reporters, Jordan and the Philippines consistently exhibited the highest means on aggression; China and Sweden exhibited the lowest means. China and Sweden also had the lowest neighborhood danger means across reporters; Italy and Kenya most often exhibited the highest means. Again, China showed the lowest parental monitoring means; Jordan and the United States most often showed the highest means. For harsh parenting, Sweden exhibited the lowest means across reporters; Kenya had the highest. Post hoc Tukey's tests confirmed that differences in means for the country comparisons highlighted here were significantly different. Correlations among the variables are shown in Table 3.

### 3.2 Main Effect of Neighborhood Danger on Child Aggression

**Figure 1:**
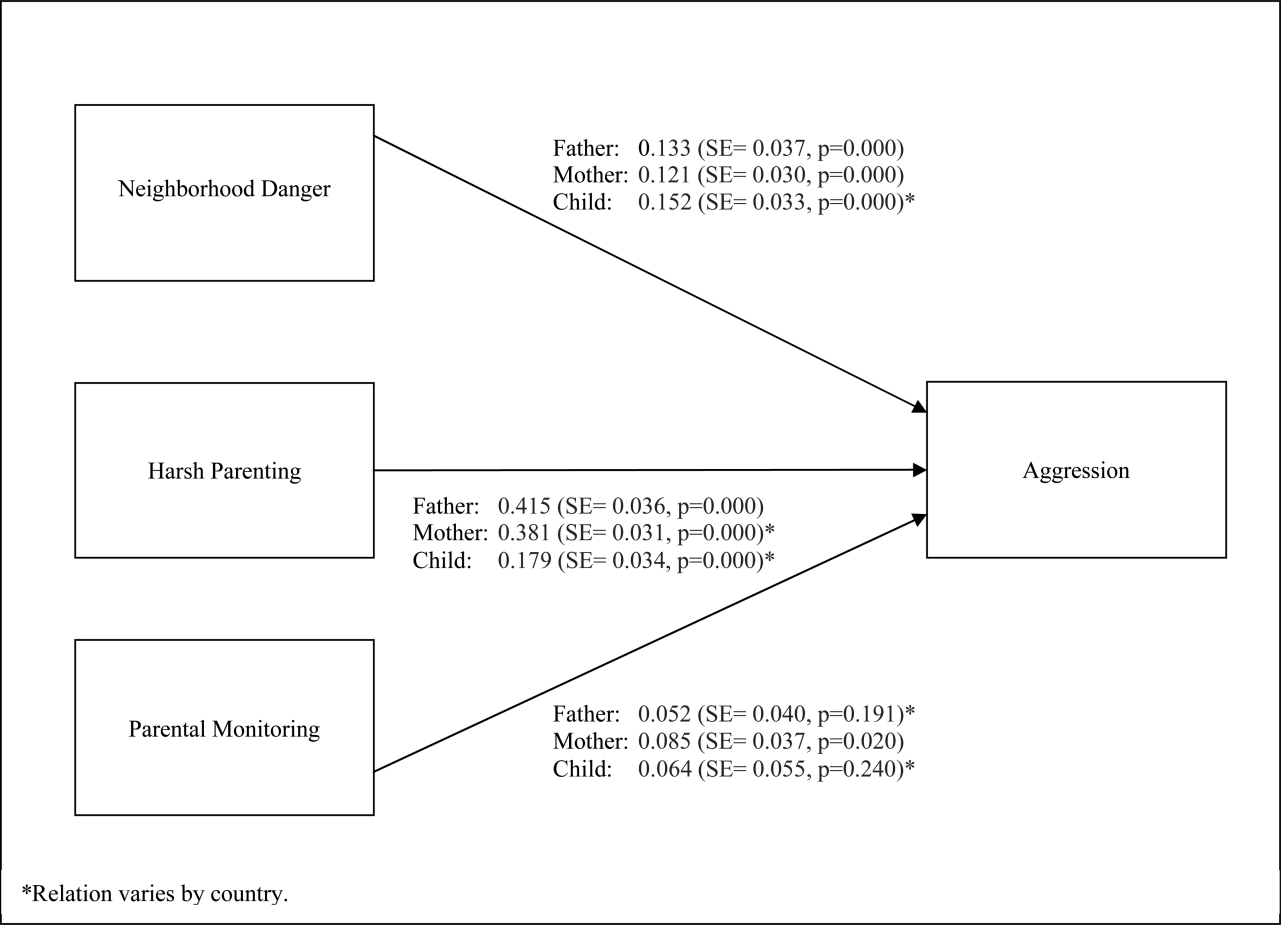
Main Effects when All Relations are Fixed across Countries

To address our first research question regarding the relation between neighborhood danger and child aggression, we estimated child aggression as a function of neighborhood danger, harsh parenting, and parental monitoring within a multi-group path model using data from fathers, mothers, and children separately. Figure 1 provides the model results when all relations are held constant across countries.

Based on father-reported data, a one standard deviation increase in grand mean-centered neighborhood danger was associated with a 0.133 standard deviation increase in grand mean-centered aggression (SE = 0.037, p = 0.000). Model fit did not improve when the relation was allowed to vary by country (chi sq = 9.644, dof = 8, p = 0.291). Using mother-reported data, a one standard deviation increase in grand mean-centered neighborhood danger was associated with a 0.121 standard deviation increase in grand mean-centered aggression (SE = 0.030, p = 0.000). Again, model fit did not improve when the relation was allowed to vary by country (chi sq = 6.987, dof = 8, p = 0.538).

Based on child-reported data, a one standard deviation increase in grand mean-centered neighborhood danger was associated with a 0.152 standard deviation increase in grand mean-centered aggression (SE = 0.033, p = 0.000). Model fit improved when the relation was allowed to vary by country (chi sq = 31.111, dof = 7, p = 0.000). A series of 28 pairwise tests comparing the relation between countries was conducted, correcting for multiple post-hoc comparisons. The tests revealed that the relation between aggression and neighborhood danger in the Philippines was statistically different from the relation in China, Colombia, Italy, Kenya, and Thailand. The relation was large and significant in the Philippines (Std Est = 0.621, SE = 0.107, p = 0.000), whereas it was non-significant in four of the comparison countries: China (Std Est = −0.029, SE = 0.078, p = 0.714), Colombia (Std Est = 0.075, SE = 0.062, p = 0.230), Kenya (Std Est = −0.057, SE = 0.073, p = 0.435), and Thailand (Std Est = 0.093, SE = 0.105, p = 0.378). In Italy, the relation was smaller relative to the Philippines but still significant (Std Est = 0.154, SE = 0.064, p = 0.016). The pairwise comparisons also revealed significant differences between the United States and two countries: China and Kenya. In the United States, a one standard deviation increase in grand mean-centered neighborhood danger was associated with a 0.303 standard deviation increase in grand mean-centered aggression (SE = 0.073, p = 0.000); the relations in China and Kenya were not statistically significant.

In summary, these models provide strong evidence of a positive relation between perceived neighborhood danger and child aggression across countries based on data reported from fathers, mothers, and children. There was some evidence, based on child-reported data, that the relation varied by country.

### 3.3 Moderation by Parental Monitoring

**Figure 2:**
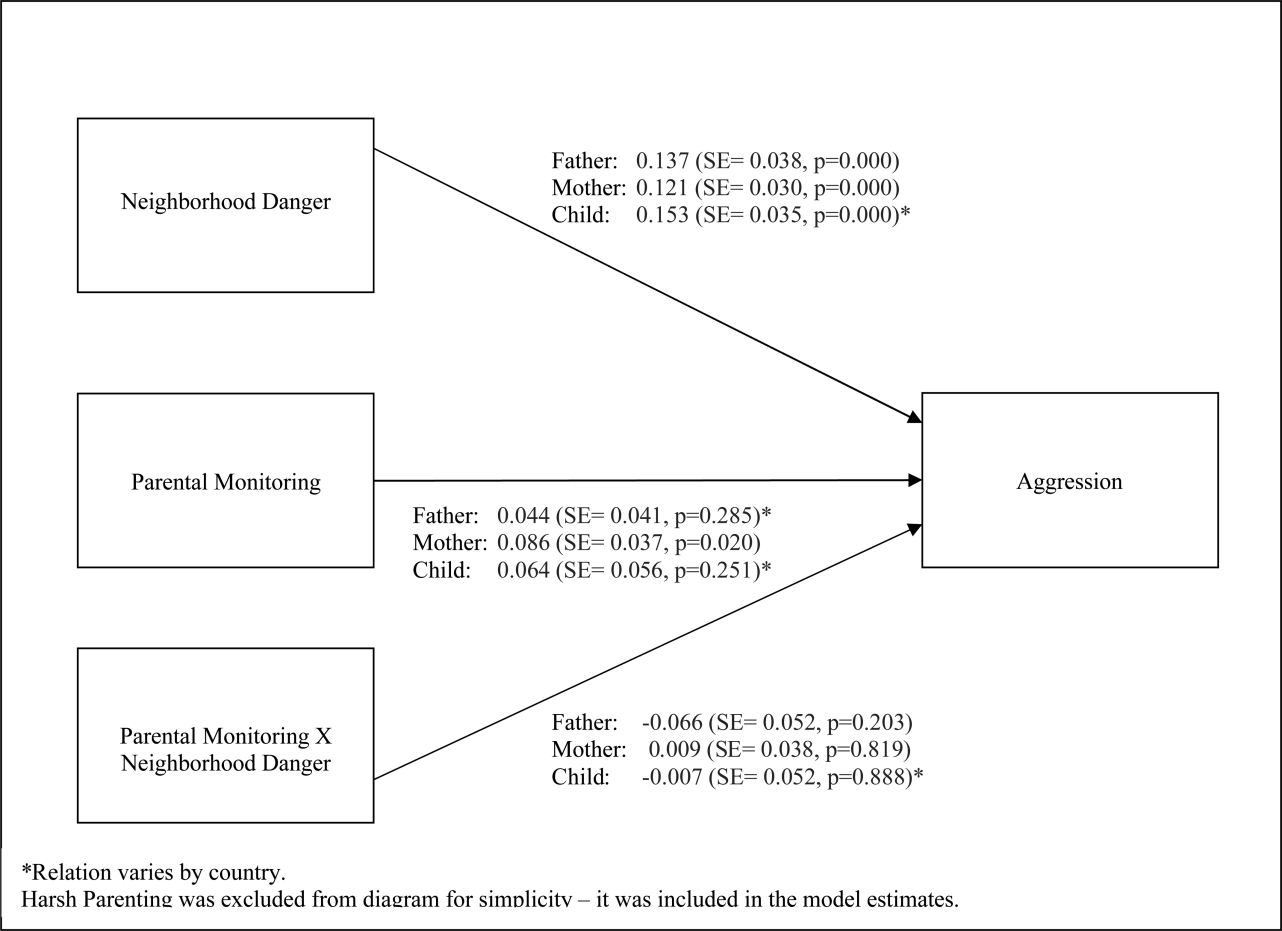
Moderation Results when All Relations are Fixed across Countries

To investigate our second research question, whether parental monitoring moderated the relation between neighborhood danger and child aggression, the interaction between danger and parental monitoring was added to the models. Figure 2 provides the moderation model results when all relations are held constant across countries.

Based on father-reported data, the interaction term was not significant (Std Est = −0.066, SE = 0.052, p = 0.203). Model fit did not improve when the relation was allowed to vary by country (chi sq = 11.380, dof = 8, p = 0.181). Based on mother-reported data, the interaction term also was not significant (Std Est = 0.009, SE = 0.038, p = 0.819). Model fit did not improve when the relation was allowed to vary by country (chi sq = 10.851, dof = 8, p = 0.210). Similarly, based on child-reported data, the interaction term was not significant (Std Est = −0.008, SE = 0.052, p = 0.888). Although model fit improved when the relation was allowed to vary by country (chi sq = 19.537, dof = 7, p = 0.007), only one pairwise difference was significant: Kenya relative to Italy. In Kenya, the interaction was positive and significant (Std Est = 0.359, SE = 0.117, p = 0.002), whereas it was negative and non-significant in Italy (Std Est = −0.226, SE = 0.132, p = 0.087). In Kenya, more neighborhood danger was associated with lower levels of child aggression (Std Est = −0.169, SE = 0.083, p = 0.042), and more parental monitoring was associated with lower child aggression (Std Est = −0.434, SE = 0.213, p = 0.041). The interaction term, however, was positive indicating that in Kenya, the lower levels of aggression associated with more neighborhood danger were lessened by more parental monitoring.

In summary, there was very little evidence across reporters that parental monitoring moderates the relation between perceived neighborhood danger and child aggression.

### 3.4 Mediation by Harsh Parenting

**Figure 3:**
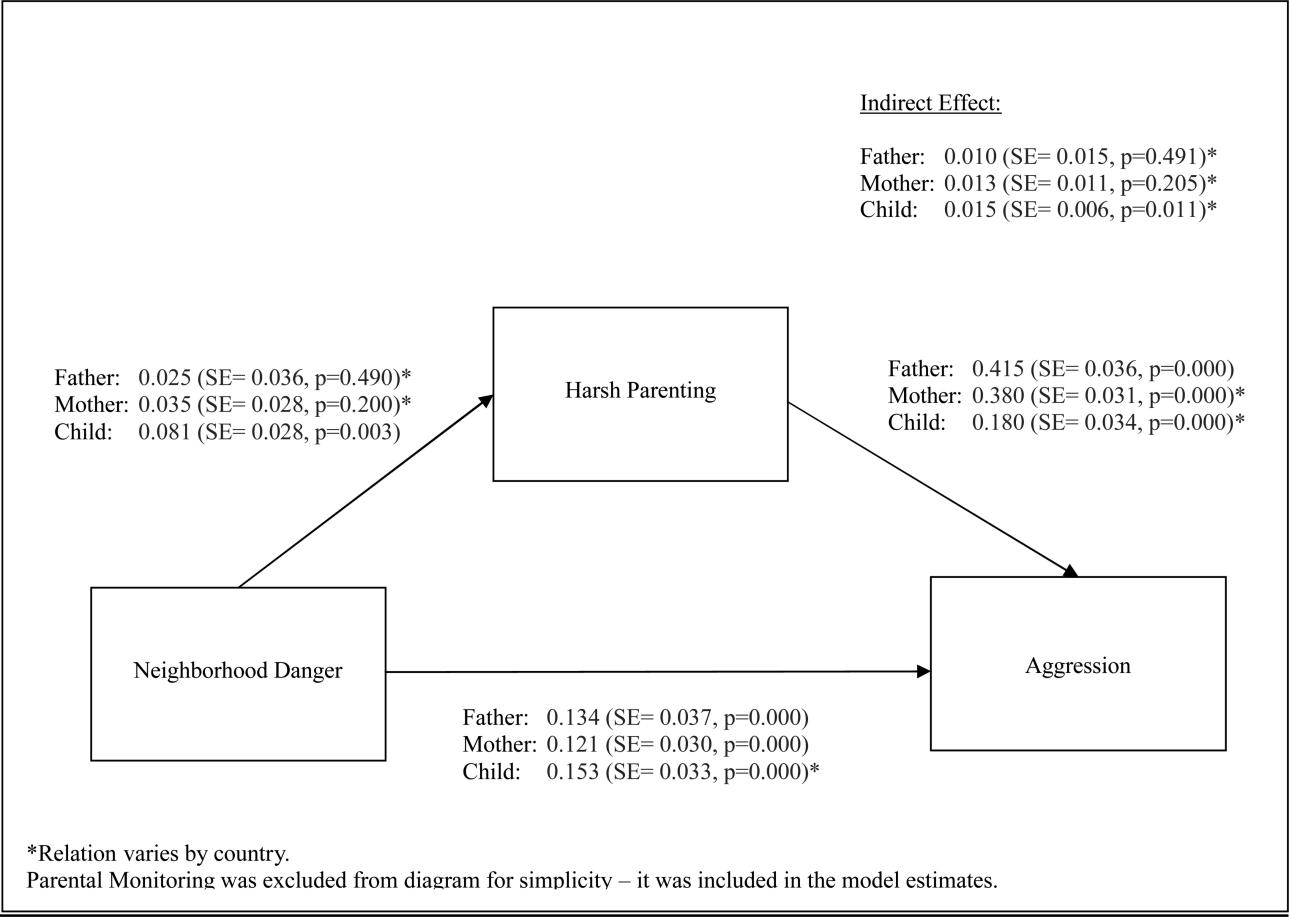
Mediation Results when All Relations are Fixed across Countries

Our third research question was whether harsh parenting mediates the link between neighborhood danger and child aggression. To test this question, we allowed neighborhood danger to predict harsh parenting. The indirect effect of neighborhood danger on aggression through harsh parenting was then estimated as the product of the harsh parenting/neighborhood danger relation and neighborhood danger/child aggression relation. Figure 3 provides the mediation model results when all relations are held constant across countries.

Using father-reported data, the relation between harsh parenting and neighborhood danger was not significant (Std Est = 0.025, SE = 0.036, p = 0.490) nor was the corresponding indirect effect of neighborhood danger on aggression (Std Est = 0.010, SE = 0.015, p = 0.491). Model fit did, however, improve when this relation was allowed to vary by country (chi sq = 26.291, dof = 8, p = 0.001). The pairwise difference tests revealed that the relation between harsh parenting and neighborhood danger in China differed from three countries (Italy, Kenya, and Sweden). In China, a one standard deviation increase in grand mean-centered neighborhood danger was associated with a 0.255 standard deviation increase in grand mean-centered harsh parenting (SE = 0.097, p = 0.008). In the comparison countries, however, an increase in neighborhood danger was associated with less harsh parenting (Italy: Std Est = −0.147, SE = 0.057, p = 0.010; Kenya: Std Est = −0.360, SE = 0.121, p = 0.003; Sweden: Std Est = −0.276, SE = 0.110, p = 0.012). The pairwise tests comparing indirect effects across countries revealed a similar pattern of differences. The positive indirect effect of neighborhood danger on child aggression through harsh parenting in China (Std Est = 0.105, SE = 0.041, p = 0.010) was significantly different from the negative indirect effects in the same three countries (Italy: Std Est = −0.061, SE = 0.023, p = 0.009; Kenya: Std Est = −0.148, SE = 0.052, p = 0.004; Sweden: Std Est = −0.114, SE = 0.046, p = 0.014).

Similarly, using mother-reported data, the relation between harsh parenting and neighborhood danger was not significant (Std Est = 0.035, SE = 0.028, p = 0.200) nor was the corresponding indirect effect (Std Est = 0.013, SE = 0.011, p = 0.205). The model fit did, however, improve when this relation was allowed to vary across countries (chi sq = 20.105, dof = 8, p = 0.010). The pairwise difference tests revealed that the relation between harsh parenting and neighborhood danger in Sweden differed from four countries (Colombia, Italy, the Philippines, and the United States). In Sweden, a one standard deviation increase in grand mean-centered neighborhood danger was associated with a 0.289 standard deviation decrease in grand mean-centered harsh parenting (SE = 0.067, p = 0.000). In Italy, the Philippines, and the United States, the relation between neighborhood danger and harsh parenting was not significant (Italy: Std Est = 0.002, SE = 0.047, p = 0.962; the Philippines: Std Est = 0.072, SE = 0.087, p = 0.411; the US: Std Est = 0.019, SE = 0.054, p = 0.721). The relation in Colombia, however, was positive and statistically significant (Std Est = 0.251, SE = 0.089, p = 0.005). The pairwise tests examining country differences in indirect effects revealed a statistically significant difference between Sweden and Colombia. In Sweden, there was evidence of a negative indirect effect of neighborhood danger on child aggression through harsh parenting (Std Est = −0.144, SE = 0.051, p = 0.005). In contrast, there was evidence of a positive indirect effect in Colombia (Std Est = 0.127, SE = 0.052, p = 0.015).

Using child-reported data, a one standard deviation increase in grand mean-centered neighborhood danger was associated with a 0.081 standard deviation increase in grand mean-centered harsh parenting (SE = 0.028, p = 0.003) with a significant and positive indirect effect (Std Est = 0.015, SE = 0.006, p = 0.011) when all relations were fixed across countries. Model fit did not improve when the harsh parenting and neighborhood danger relation was allowed to vary by country (chi sq = 9.509, dof = 7, p = 0.218). As discussed previously, the fit did, however, improve when the relation between neighborhood danger and child aggression was allowed to vary by country, yielding variation in the indirect effect across countries. The series of pairwise tests examining differences in indirect effects between countries revealed no statistically significant differences.

In summary, using child reports, we found support for our hypothesis that harsh parenting would mediate the link between perceived neighborhood danger and child aggression. Using fathers’ and mothers’ reports, the mediating role of harsh parenting varied across countries.

## 4. Discussion

This study was designed to examine the relation of perceived neighborhood danger with child aggression across nine countries. The relation between exposure to neighborhood danger and child aggressive behavior has been well documented in the United States, but this is the first study to examine this relation across such a wide range of diverse countries and informants. This study further tests this relation by examining whether parental monitoring moderates and whether harsh parenting mediates the link between neighborhood danger and child aggression. In a departure from many single-informant studies and in order to strengthen our study, we included mother, father, and child perceptions of neighborhood danger, parental monitoring, and child adjustment, and mother and father reports of harsh parenting. In doing so, we found that greater neighborhood danger was related to more child aggression, and parental monitoring did not moderate this relation. However, the role of harsh parenting as a mediator was less consistent across countries and reporters.

In each country, we asked mothers, fathers, and children to report on their perceptions of neighborhood danger and child aggression. Our first hypothesis, that neighborhood danger would be related to child aggression, was supported in all nine countries for mother and father reports. This link also was found for child reports in five of the nine countries. Although research regarding childhood exposure to neighborhood danger in sites outside the United States is not as extensive as the work done in the United States, other studies of children exposed to violence in countries involved in armed conflict document similar effects and make our result not surprising [49-51]. Our work extends previous research, which has focused largely on links between exposure to political violence and children's adjustment to links between more general perceptions of neighborhood danger and children's aggression. It is interesting that the links between neighborhood danger and children's aggression did not vary across countries when mothers and fathers reported on the constructs but that some differences emerged when children reported on their own perceptions of neighborhood danger and aggression. It is possible that children are exposed to more or less danger in their neighborhoods than their parents perceive them to be, which supports the importance of including children's perceptions in multi-informant designs.

Our second hypothesis, that parental monitoring would moderate the relation between perceived neighborhood danger and child aggression, was not supported. Given that recent conceptualizations of parental monitoring highlight a range of behaviors and knowledge that constitute different aspects of monitoring [52, 53], we incorporated parents’ attempts to limit their children's behaviors and parents’ attempts to gain knowledge about different aspects of their children's lives into our construct measuring parental monitoring. With the exception of a difference between two countries based on children's reports, we found no evidence that relations between neighborhood danger and child aggression were moderated by these aspects of parental monitoring.

Although this general lack of evidence for moderation did not support our hypothesis, it is not entirely inconsistent with previous research, in which findings have been mixed, even within the United States [54]. There are many reasons that parental monitoring may not moderate relations between neighborhood danger and child aggression in our diverse sample of countries. First, given the range of ways in which parental monitoring has been conceptualized and operationalized, it is possible that in previous studies that found moderation, parental monitoring was capturing a set of parenting variables that were not measured in the current study. As Stattin and Kerr [53] argued, parental monitoring may be less about parental restriction of activities and more about child disclosure of information. Our measure of parental monitoring did not assess child disclosure of information but instead focused on parents’ attempts to gain knowledge and impose limitations. Furthermore, although strong evidence points to the need to include parent and child perceptions of neighborhood danger [25], which we did in this study, we did not include any objective measure of neighborhood danger such as crime statistics. Even children who experience high levels of danger in their neighborhood may feel protected, perhaps by living in a more secure part of the neighborhood, and have little direct experience with violence. It is also possible that parental monitoring moderates the association between neighborhood danger and child aggression primarily in contexts that are relatively low in danger (when monitoring may be able to mitigate some low-level risks) or in contexts that are relatively high in danger (when children would be most vulnerable). An important direction for future research will be to examine which aspects of parental monitoring moderate links between neighborhood danger and child aggression and under what conditions of neighborhood danger, including proximity, frequency, and severity. Furthermore, it is possible that changes in protective parenting behavior through increased parental monitoring are a reaction to an experience of neighborhood danger, but one that comes too late after a child is aware of or has experienced traumatic events to make a difference in the outcome as it is defined by child aggression. Future research should aim to gather more specifics about the frequency and duration of exposure to neighborhood danger and measure changes in parental monitoring over time.

We found inconsistent support for our third hypothesis, that harsh parenting would mediate the relation between perceived neighborhood danger and child aggression. Children's reports showed a consistent pattern of mediation across countries, with more neighborhood danger predicting harsher parenting, which in turn predicted more child aggression. The patterns of mediation differed across countries in the father- and mother-reported models. In the mother-reported models, we urge caution in the interpretation of the findings because the country differences were driven by comparisons of Sweden with other countries, and reports of harsh parenting in Sweden were very low. Corporal punishment was outlawed in Sweden in 1979, and many of the items in our harsh parenting measure reflected different forms of corporal punishment. Previous studies have documented relations between harsh parenting and child aggression across multiple cultural contexts [18, 21], and the present study also found evidence of that direct association. However, it is not known from the current study exactly in what way and under what conditions or what dosage exposure to neighborhood danger has an effect on harsh parenting; these would be other important questions for future research.

### 4.2 Limitations and Future Directions

Although our study advanced knowledge of neighborhood danger in a cross-national sample, several limitations should be noted. First, the data are cross-sectional, so we cannot draw conclusions regarding the direction of effects. In particular, children who behave aggressively elicit harsher parenting than do children without aggressive behavior problems [55]. Similarly, because we did not measure changes in levels of perceptions of neighborhood danger or changes in monitoring over time or developmental period, we were unable to determine if increased parental monitoring was simply unable to attenuate the relation between neighborhood danger and child aggression because the monitoring came into play too late. Further research should include longitudinal data that are able to unpack the specific elements of parental monitoring that may be responsible for relations between exposure to community violence and child adjustment. Because a consistent link between neighborhood danger and child aggression was found across countries, yet the parenting variables did not consistently alter or explain this relation, this leaves open the question of which other factors within or outside the family might moderate or mediate the link between perceptions of neighborhood danger and child aggression. In addition, future research that delves into other aspects of children's experiences might be able to elucidate more fully why children's perceptions of neighborhood danger were not related to children's reports of their own aggression in China, Colombia, Kenya, and Thailand as they were in the other five countries and according to mothers’ and fathers’ reports in all nine countries.

### 4.3 Conclusion

To advance understanding of complex relations between exposure to neighborhood danger and child aggression, we collected data from 1,293 families in nine countries. Our work largely supported previous research on primarily American samples and documented a relation between perceived neighborhood danger and child aggression across all nine countries using reports from mothers and fathers, and in five of the nine countries using reports from children. In contrast to previous research, however, our hypotheses about the moderating role of parental monitoring and the mediating role of harsh parenting were inconsistent. Much of the research about parental monitoring tells us there is still much to learn about the interaction between exposure to violence during childhood, and what specific aspects of parental monitoring are important; we only measured two: parents’ attempts to know what their children were doing and parents’ attempts to limit their children's behavior. Research that also includes measures of children's disclosure of behavior and activities could strengthen future studies. Nevertheless, the overall take-home message is clear: Mothers’, fathers’, and children's perceptions of higher levels of neighborhood danger are related to higher levels of child aggression in diverse countries around the world. Questions about how and under what conditions remain to be answered.

## Figures and Tables

**Table 1:** Demographic Descriptive Statistics by Country: Means, (Standard Deviations), and sample size.

	Male	Age	Income	Education
China	0.48 (0.50) n=242	10.23 (0.41) n=242	5.85 (3.27) n=208	13.57 (2.75) n=242
Colombia	0.44 (0.50) n=108	10.24 (0.61) n=108	3.96 (2.95) n=102	11.53 (4.90) n=108
Italy	0.50 (0.50) n=209	10.93 (0.63) n=209	4.52 (2.28) n=202	12.95 (4.09) n=209
Jordan	0.53 (0.50) n=114	10.83 (0.35) n=114	1.75 (1.27) n=114	14.13 (2.36) n=114
Kenya	0.40 (0.49) n=100	10.80 (0.86) n=100	2.17 (1.66) n=98	12.67 (3.11) n=100
Philippines	0.51 (0.50) n=120	10.52 (0.41) n=120	5.23 (2.90) n=108	14.48 (3.30) n=120
Sweden	0.51 (0.50) n=103	10.13 (0.35) n=103	7.96 (2.35) n=100	14.66 (2.65) n=103
Thailand	0.51 (0.50) n=120	10.78 (0.63) n=120	3.75 (2.18) n=119	13.20 (4.27) n=120
US	0.51 (0.50) n=315	11.13 (0.63) n=315	6.05 (2.97) n=287	14.24 (3.93) n=315

**Table 2:** Descriptive Statistics by Country: Means, (Standard Deviations), sample size, and [alpha coefficient].

	Child Aggression			Neighborhood Danger			Parental Monitoring			Harsh Parenting	
	Father	Mother	Child	Father	Mother	Child	Father	Mother	Child	Father	Mother
China	0.27 (0.22) n=214 [0.84]	0.28 (0.21) n=217 [0.80]	0.25 (0.21) n=218 [0.80]	0.18 (0.39) n=214 [0.83]	0.16 (0.45) n=216 [0.90]	0.21 (0.43) n=218 [0.72]	−0.50 (0.72) n=214 [0.88]	−0.80 (0.70) n=217 [0.83]	−0.70 (0.60) n=218 [0.80]	0.12 (0.17) n=215 [0.68]	0.13 (0.20) n=217 [0.74]
Colombia	0.51 (0.31) n=95 [0.85]	0.52 (0.32) n=100 [0.87]	0.30 (0.21) n=100 [0.71]	0.72 (0.84) n=95 [0.88]	0.70 (0.94) n=100 [0.92]	0.61 (0.72) n=100 [0.75]	0.26 (0.59) n=95 [0.84]	0.32 (0.50) n=100 [0.79]	0.26 (0.50) n=100 [0.73]	0.19 (0.17) n=95 [0.68]	0.24 (0.22) n=100 [0.79]
Italy	0.43 (0.27) n=152 [0.83]	0.49 (0.29) n=194 [0.83]	0.40 (0.24) n=194 [0.76]	0.86 (0.80) n=152 [0.87]	0.96 (0.90) n=194 [0.88]	0.56 (0.62) n=194 [0.72]	−0.00 (0.63) n=152 [0.85]	0.25 (0.47) n=194 [0.77]	0.20 (0.48) n=194 [0.73]	0.18 (0.17) n=152 [0.69]	0.26 (0.18) n=194 [0.59]
Jordan	0.40 (0.30) n=109 [0.88]	0.44 (0.30) n=112 [0.88]	0.52 (0.33) n=112 [0.87]	0.29 (0.44) n=108 [0.68]	0.36 (0.48) n=112 [0.72]	0.43 (0.50) n=112 [0.64]	0.13 (0.67) n=109 [0.88]	0.30 (0.39) n=112 [0.73]	0.31 (0.60) n=112 [0.87]	0.23 (0.22) n=109 [0.73]	0.26 (0.24) n=112 [0.73]
Kenya	0.34 (0.26) n=94 [0.83]	0.34 (0.21) n=95 [0.71]	0.38 (0.22) n=95 [0.73]	0.69 (0.65) n=94 [0.86]	0.73 (0.66) n=95 [0.84]	0.80 (0.68) n=95 [0.83]	0.04 (0.70) n=94 [0.89]	−0.10 (0.56) n=95 [0.78]	0.14 (0.59) n=95 [0.86]	0.36 (0.28) n=94 [0.76]	0.47 (0.31) n=95 [0.78]
Philippines	0.47 (0.28) n=79 [0.86]	0.53 (0.33) n=100 [0.89]	0.52 (0.32) n=103 [0.88]	0.59 (0.66) n=79 [0.82]	0.80 (0.81) n=100 [0.86]	0.65 (0.56) n=103 [0.61]	0.16 (0.54) n=79 [0.82]	0.12 (0.60) n=100 [0.86]	0.15 (0.52) n=103 [0.78]	0.18 (0.20) n=78 [0.71]	0.22 (0.20) n=100 [0.70]
Sweden	0.18 (0.17) n=72 [0.80]	0.20 (0.17) n=95 [0.77]	0.27 (0.2) n=98 [0.79]	0.07 (0.18) n=72 [0.66]	0.08 (0.21) n=95 [0.60]	na na n=0 na	0.16 (0.38) n=72 [0.74]	0.05 (0.48) n=95 [0.82]	−0.2 (0.51) n=98 [0.79]	0.08 (0.09) n=72 [0.23]	0.07 (0.08) n=94 [0.22]
Thailand	0.33 (0.26) n=82 [0.87]	0.31 (0.23) n=100 [0.85]	0.44 (0.29) n=101 [0.85]	0.56 (0.56) n=82 [0.77]	0.57 (0.61) n=100 [0.79]	0.58 (0.58) n=101 [0.63]	0.04 (0.65) n=82 [0.89]	−0.10 (0.59) n=100 [0.84]	0.11 (0.51) n=101 [0.75]	0.12 (0.17) n=82 [0.67]	0.13 (0.18) n=100 [0.70]
US	0.30 (0.24) n=183 [0.83]	0.33 (0.33) n=273 [0.91]	0.34 (0.29) n=272 [0.87]	0.24 (0.45) n=182 [0.82]	0.28 (0.52) n=273 [0.82]	0.38 (0.56) n=272 [0.77]	0.27 (0.60) n=182 [0.85]	0.32 (0.51) n=273 [0.83]	0.16 (0.51) n=272 [0.75]	0.08 (0.11) n=183 [0.42]	0.12 (0.15) n=272 [0.55]

**Table 3:** Correlation Coefficients (p-values and sample sizes)

	Child Aggression			Neighborhood Danger			Parental Monitoring			Harsh Parenting		
	Father	Mother	Child	Father	Mother	Child	Father	Mother	Child	Father	Mother	Average
Father-Reported Child Aggression	1.00 1080	0.55 <.01 1072	0.34 <.01 1079	0.21 <.01 1077	0.14 <.01 1071	0.17 <.01 1007	0.05 0.11 1078	0.15 <.01 1072	0.16 <.01 1079	0.40 <.01 1079	0.29 <.01 1071	0.40 <.01 1080
Mother-Reported Child Aggression	0.55 <.01 1072	1.00 1286	0.34 <.01 1282	0.18 <.01 1070	0.24 <.01 1285	0.18 <.01 1187	0.07 0.02 1071	0.16 <.01 1286	0.13 <.01 1282	0.24 <.01 1072	0.42 <.01 1283	0.40 <.01 1285
Child-Reported Child Aggression	0.34 <.01 1079	0.34 <.01 1282	1.00 1293	0.09 0.00 1077	0.09 0.00 1281	0.24 <.01 1195	0.07 0.03 1078	0.10 0.00 1282	0.16 <.01 1293	0.19 <.01 1079	0.22 <.01 1280	0.24 <.01 1289
Father-Reported Neighborhood Danger	0.21 <.01 1077	0.18 <.01 1070	0.09 0.00 1077	1.00 1078	0.62 <.01 1070	0.32 <.01 1005	0.09 0.00 1077	0.13 <.01 1070	0.20 <.01 1077	0.10 0.00 1077	0.15 <.01 1069	0.15 <.01 1078
Mother-Reported Neighborhood Danger	0.14 <.01 1071	0.24 <.01 1285	0.09 0.00 1281	0.62 <.01 1070	1.00 1285	0.34 <.01 1186	0.11 0.00 1070	0.12 <.01 1285	0.16 <.01 1281	0.07 0.02 1071	0.17 <.01 1282	0.16 <.01 1284
Child-Reported Neighborhood Danger	0.17 <.01 1007	0.18 <.01 1187	0.24 <.01 1195	0.32 <.01 1005	0.34 <.01 1186	1.00 1195	0.16 <.01 1006	0.12 <.01 1187	0.12 <.01 1195	0.12 0.00 1007	0.20 <.01 1186	0.18 <.01 1194
Father-Reported Parental Monitoring	0.05 0.11 1078	0.07 0.02 1071	0.07 0.03 1078	0.09 0.00 1077	0.11 0.00 1070	0.16 <.01 1006	1.00 1079	0.40 <.01 1071	0.28 <.01 1078	−0.01 0.66 1078	0.03 0.34 1071	0.01 0.76 1079
Mother-Reported Parental Monitoring	0.15 <.01 1072	0.16 <.01 1286	0.10 0.00 1282	0.13 <.01 1070	0.12 <.01 1285	0.12 <.01 1187	0.40 <.01 1071	1.00 1286	0.41 <.01 1282	0.05 0.11 1072	0.08 0.00 1283	0.08 0.01 1285
Child-Reported Parental Monitoring	0.16 <.01 1079	0.13 <.01 1282	0.16 <.01 1293	0.20 <.01 1077	0.16 <.01 1281	0.12 <.01 1195	0.28 <.01 1078	0.41 <.01 1282	1.00 1293	0.13 <.01 1079	0.15 <.01 1280	0.16 <.01 1289
Father-Reported Harsh Parenting	0.40 <.01 1079	0.24 <.01 1072	0.19 <.01 1079	0.10 0.00 1077	0.07 0.02 1071	0.12 0.00 1007	−0.01 0.66 1078	0.05 0.11 1072	0.13 <.01 1079	1.00 1080	0.45 <.01 1071	0.83 <.01 1080
Mother-Reported Harsh Parenting	0.29 <.01 1071	0.42 <.01 1283	0.22 <.01 1280	0.15 <.01 1069	0.17 <.01 1282	0.20 <.01 1186	0.03 0.34 1071	0.08 0.00 1283	0.15 <.01 1280	0.45 <.01 1071	1.00 1284	0.89 <.01 1284
Harsh Parenting Father & Mother Average)	0.40 <.01 1080	0.40 <.01 1285	0.24 <.01 1289	0.15 <.01 1078	0.16 <.01 1284	0.18 <.01 1194	0.01 0.76 1079	0.08 0.01 1285	0.16 <.01 1289	0.83 <.01 1080	0.89 <.01 1284	1.00 1293
